# Left ventricular deformation mechanics over time in patients with thalassemia major with and without iron overload

**DOI:** 10.1186/s12872-021-01897-8

**Published:** 2021-02-09

**Authors:** Michael J. Bonios, Epameinontas Fountas, Polyxeni Delaporta, Stamatis Kyrzopoulos, Antonis Kattamis, Stamatis N. Adamopoulos, Dimitris Tsiapras

**Affiliations:** 1 Heart Failure and Transplant Unit, Onassis Cardiac Surgery Center, Athens, Greece; 2 Noninvasive Department of Cardiology, Onassis Cardiac Surgery Center, 356 Sygrou Av, 176 74 Athens, Greece; 3 Hematology/Oncology Unit, First Department of Pediatrics, National and Kapodistrian University of Athens, Athens, Greece

**Keywords:** Thalassemia major, Echocardiography, Strain, Iron overload

## Abstract

**Background:**

Myocardial iron overload in patients with thalassemia major (TM) is one of the most important complications. The purpose of the study was to identify advanced echocardiography parameters for early identification of myocardial dysfunction during follow-up of patients with TM.

**Methods:**

Forty TM patients who were 41 ± 5 years old were included in the study and divided into two groups according to cardiac magnetic resonance T2* results (Group 1: Τ2* > 25 ms, Group 2: Τ2* ≤ 25 ms). Liver T2* parameters were also measured. Conventional and deformational echocardiographic parameters were measured at baseline and approximately 2 years later.

**Results:**

Thirty-two patients had Τ2* = 34 ± 4 ms (Group 1), and 8 had Τ2* = 17 ± 9 ms (Group 2). Blood consumption was 185 ± 60 and 199 ± 37 ml/kg/yr (*p* = 0.64), and liver T2* was 4 ± 5 and 17 ± 21 ms (*p* = 0.01) in Groups 1 and 2, respectively. At baseline, Group 1 had better left ventricular global longitudinal strain (GLS) (− 22 ± 3 vs. − 18 ± 5, *p* = 0.01) and similar left ventricular ejection fraction (LVEF) (62 ± 5% vs. 58 ± 10%, *p* = 0.086) than Group 2. At the 28 ± 11-month follow-up, LVEF, GLS, and T2* values in Group 1 (63 ± 3%, − 21 ± 3%, 34 ± 4 ms) and Group 2 (56 ± 11%, − 17 ± 4%, 17 ± 9 ms) did not change significantly compared to their corresponding baseline values. In 8 patients from Group 1, a worsening (> 15%) in LS (*p* = 0.001) was detected during follow-up, with a marginal reduction in LVEF.

**Conclusions:**

GLS seems to be an efficient echocardiographic parameter for detecting hemochromatosis-related cardiac dysfunction earlier than LVEF. It also seems to be affected by other factors (free radical oxygen, immunogenetic mechanisms or viral infections) in a minority of patients, underscoring the multifactorial etiology of cardiomyopathy.

## Background

Thalassemia major (TM) is one of the most common inherited hemoglobin disorders. Ineffective erythropoiesis results in hemolytic anemia, and the patient is in need of lifelong transfusion therapy that ultimately leads to iron overload. Despite advances in the field of iron chelator therapies for TM, iron continues to accumulate in heart tissue, and subsequent cardiomyopathy remains the leading cause of death for these patients [1–3].

The pathophysiology of the cardiomyopathy that develops in the modern era of iron chelator therapies is more complicated. Even though iron overload is still considered the leading cause of the occurrence of heart failure in patients with TM, the production of free radical oxygen, immunogenetic mechanisms and viral infections are being increasingly recognized [2, 4, 5].

The complex etiopathogenetic milieu of thalassemia cardiomyopathy requires identifying the dysfunctional myocardium at an early stage to make the most of the early implementation of medical therapies. While cardiac magnetic resonance imaging (MRI) provides the ability to directly and noninvasively measure cardiac iron [6, 7], echocardiography is still the first-line imaging tool for the assessment of myocardial function. Notably, however, conventional echocardiography parameters may still be within the normal range before the development of overt heart dysfunction. Newer echocardiographic techniques focusing on the analysis of myocardial deformation have been proven to be potentially useful tools for the early identification of myocardial dysfunction [8].

The purpose of our study was to evaluate the changes in left ventricular global longitudinal strain and circumferential strain in patients with thalassemia major with and without myocardial iron overload.

## Methods

We retrospectively studied 70 consecutive patients diagnosed with TM who were receiving blood transfusions and chelation therapy. Patients were eligible for enrollment when they fulfilled all the following criteria: (a) cardiac MRI for T2* measurement at the beginning of the study and during the follow-up period and (b) echocardiographic study at the initiation of the study and during the follow-up period. Patients were included in the study if their initial LVEF was more than 50%, (c) their transthoracic echocardiographic images for the measurement of left ventricular longitudinal and circumferential strain were of adequate quality, and (d) they had undergone a follow-up period between 12 and 36 months.

Patients with heart failure, valvulopathy, or history of myocardial infarction were not included in the study.

### Echocardiography

Patients were studied in the left lateral decubitus position with a commercially available system (GE E9, Horten, Norway) coupled with a 3.5 MHz (M3S) transducer. Analysis was performed offline.

#### Echocardiographic study protocol

Cardiac MRI was performed no later than 1 month after the echocardiography study. Echocardiographic studies included complete 2D and Doppler examinations. Assessment of LVEF was performed using apical 4- and 2-chamber views. Doppler evaluation included the assessment of mitral inflow velocities. The mitral inflow parameters evaluated were early mitral inflow velocity (E-wave) and late or atrial mitral inflow velocity (a-wave). Peak right ventricular systolic myocardial velocity (RVSm) was obtained by placing the sample volume of tissue Doppler imaging at the lateral tricuspid valve annulus. All measurements were performed by following the current European Society of Echocardiography guidelines [9, 10].

#### Deformational echocardiographic analysis

For both global longitudinal strain (GLS) and circumferential strain (CS) measurements, analysis was performed with dedicated software (EchoPAC v11, General Electric Medical Systems).

#### Longitudinal strain

Grayscale two-dimensional apical images of the LV (4-, 2-, and 3-chamber views) were obtained, and global longitudinal strain (GLS) analysis of the LV was performed by speckle-tracking imaging [9, 10]. Three consecutive beats in each view were stored digitally for offline analysis. Mean values were calculated for all measured parameters. The frame rate was set between 50 and 100 frames/s, sector width was set as narrow as possible, and gain settings were optimized. For each view, three consecutive beats were analyzed, and mean values were calculated for all parameters derived. After the cardiac cycle was selected, the software prompted the operator to apply a region of interest in a ‘‘click-to-point approach'' to delineate the endocardium. Subsequently, the software automatically defined an epicardial and midmyocardial line and processed all frames of the selected cardiac cycle. Global longitudinal strain (GLS) was calculated as the average LS from all segments generated by software analysis of the 3 apical views.

Additionally, we separately studied non-iron–overloaded TM patients who demonstrated a relative percentage of worsening in the GLS > 15% during the follow-up period. We considered the relative percentage reduction in GLS > 15% to be a clinically meaningful reduction, similar to the cases of oncology patients where the changes in GLS are used for the early identification of myocardial damage following the administration of agents with potential cardiotoxic properties [11].

#### Circumferential strain

Circumferential strain (CS) of the mid-LV was calculated using the short-axis view at the level of the papillary muscles. Peak CS was defined as the average CS of all 6 segments (generated as previously described) in the short-axis view.

### Magnetic resonance imaging for cardiac and liver iron measurement

Magnetic resonance imaging examinations were performed with a 1.5-T scanner (Symphony, Siemens, Erlangen, Germany). The scans included measurements of the liver T2* and myocardial T2* values. The T2* of the heart was assessed by a cardiac-gated single breath-hold multiecho technique (FOV, 400 mm; TR, 135 ms; TE, 2.6–22.3 ms (8 echo times); flip angle, 20; slice thickness, 10 mm; matrix, 192 × 75; number of averages, 1; bandwidth in Hz/pixel, 810).

### Statistical analysis

Values are reported as the mean ± SD. The paired *t*-test was used for intragroup comparisons of the echocardiography measurements at baseline and during follow-up. Independent-sample *t*-tests were used to compare baseline parameters of the TMio and Group 2 patients. For values with a nonnormal distribution, the Mann–Whitney U test was used. The χ2 test was used to compare the noncontinuous characteristics between the 2 groups. Spearman’s correlation coefficient was used to assess correlations of the measured parameters. The statistical software package SPSS for Windows was used for the analysis (SPSS 18 Inc., Chicago, IL, USA). A *p*-value < 0.05 was considered statistically significant.

## Results

**Fig. 1:**
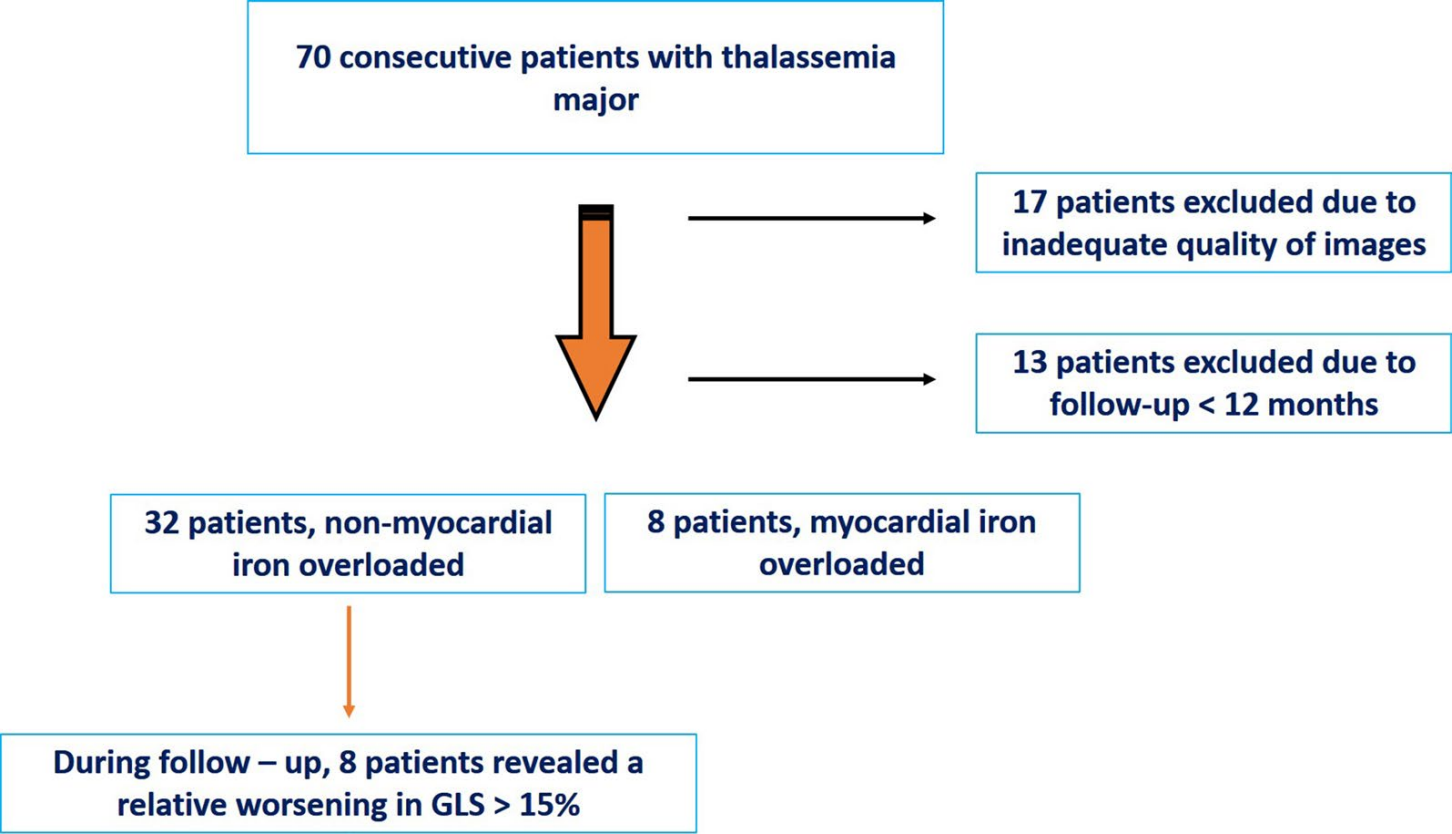
Flow diagram of patients included in the study

Out of the 70 patients with TM disease who we identified in our database, 40 fulfilled the criteria for inclusion in our study (Fig. 1). Seventeen patients were excluded due to inadequate image quality, and 13 patients were excluded because they had a follow-up period of less than 12 months.

### Patient characteristics

The age of the patients was 41 ± 5 years, and 22 were males. Chelation therapy consisted of desferrioxamine (n = 7), defersirox (n = 14), deferiprone (n = 4), desferrioxamine + defersirox (n = 2), and desferrioxamine + deferiprone (n = 13) (Table 1).

**Table 1:** Baseline profile of the Thalassaemia Major patients group according to CMR-T2* values

	Group1 (*n* = 32)	Group2 (*n* = 8)	*P*
Age	42 ± 4	39 ± 8	0.45
BSA (m^2^)	1.69 ± 0.12	1.67 ± 0.23	0.75
Pure RBC (ml/kg/yr)	185 ± 60	199 ± 37	0.64
Serum ferritin (mg/L)	1217 ± 1804	3663 ± 4204	0.02
Cardiac T2* (ms)	33 ± 3	16 ± 9	0.01
Liver T2* (ms)	4 ± 5	17 ± 21	0.01
Splenectomy (n)	12	2	0.68
Diabetes (n)	7	0	0.18
Medication (n)			0.02
*Chelators*			
Deferoxamine	5	2	
Deferasirox	13	1	
Deferiprone	4	0	
Deferoxamine + Deferasirox	0	2	
Deferoxamine + Deferiprone	10	3	
Beta-blockers	4	2	0.28
ACEI/ARB	2	1	0.46

*ACEI* ACE inhibitors, *ARB* angiotensin receptor blockers, *BSA* body surface area, *CMR* Cardiac Magnetic Resonance, *LIC* liver iron concentration, *RBC* red blood cells

Eight TM patients were identified as iron overloaded based on cardiac MRI (T2* ≤ 25 ms, Group 2) [12, 13]. In this group, 4 patients had marginal T2* (20–25 ms), and 4 patients had abnormal T2* < 20 ms. The rest of the patients (n = 32) had a T2* > 25 ms and composed the non-iron–overloaded group (Group 1). The baseline characteristics of the two patient groups are shown in Table 1. Group 2 patients had significantly higher mean serum ferritin levels and a higher liver iron load than group 1 patients.

#### Conventional echocardiography and deformational parameters at baseline

Analysis of strain parameters was performed by one experienced operator. Group 1 had similar LVEF to Group 2 patients. Additionally, Group 1 had significantly better GLS values than Group 2. No differences in CS values were observed between the two study groups (Table 2).

**Table 2:** Echocardiographic and MRI data at baseline and during follow-up

	T2* > 25 ms (n = 32) Group1		*P*	T2* ≤ 25 ms (n = 8) Group2		*P*
	Βaseline	Follow-up		Baseline	Follow-up	
*Echocardiographic data*						
Conventional						
LVEF (%)	62 ± 5	63 ± 3	0.60	58 ± 10	56 ± 11	0.17
LVEDD (mm)	49 ± 4	48 ± 4	0.10	49 ± 4	48 ± 4	0.36
LVESD (mm)	31 ± 4	30 ± 4	0.72	31 ± 6	32 ± 6	0.10
IVS (mm)	8.6 ± 0.9	8.7 ± 0.9	0.50	8.1 ± 0.9	8.5 ± 0.9	0.08
E/A	1.5 ± 0.6	1.4 ± 0.5	0.93	1.5 ± 0.6	1.4 ± 0.3	0.68
RVSm (cm/sec)	15.0 ± 1.9	15.1 ± 1.8	0.84	14.8 ± 2.3	14.6 ± 1.7	0.81
Deformational						
Circumferential strain (%)	− 22 ± 5	− 22 ± 5	0.71	− 20 ± 6	− 18 ± 6	0.22
Circumferential strain rate	− 1.4 ± 0.9	− 1.4 ± 0.4	0.65	− 1.2 ± 0.4	− 1.2 ± 0.5	0.64
Global Longitudinal strain (%)	− 22 ± 3	− 21 ± 3	0.24	− 18 ± 5 *	− 17 ± 4	0.38
Mean Longitudinal strain rate	− 1.2 ± 0.3	− 1.2 ± 0.2	0.37	− 1.0 ± 0.3 **	− 1.0 ± 0.2	0.68
T2* (msec)	33 ± 3	34 ± 4	0.52	16 ± 9 ***	17 ± 9	0.55

*LVEF* left ventricular ejection fraction, *LVEDD* left ventricular end-diastolic diameter, *LVESD* left ventricular end-systolic diameter, *IVS* intraventricular septum thickness, *E/A* early to atrial transmitral flow ratio, *RVSm* right ventricular basal myocardial systolic velocity

^*^p = 0.01; **p = 0.04; ***p = 0.01, all compared to the baseline values of the Group1

#### Mid-term follow-up of patients with TM

The mean follow-up of TM patients was 29 ± 11 months (range 12–36 months). Patient chelation therapy was adjusted by the attending physicians. There was no significant change in either conventional or deformational parameters during the follow-up period for either group of patients. Interestingly, during the follow-up period, we detected no significant changes in serum ferritin or T2* signals for either group of patients (Table 2).

**Fig. 2:**
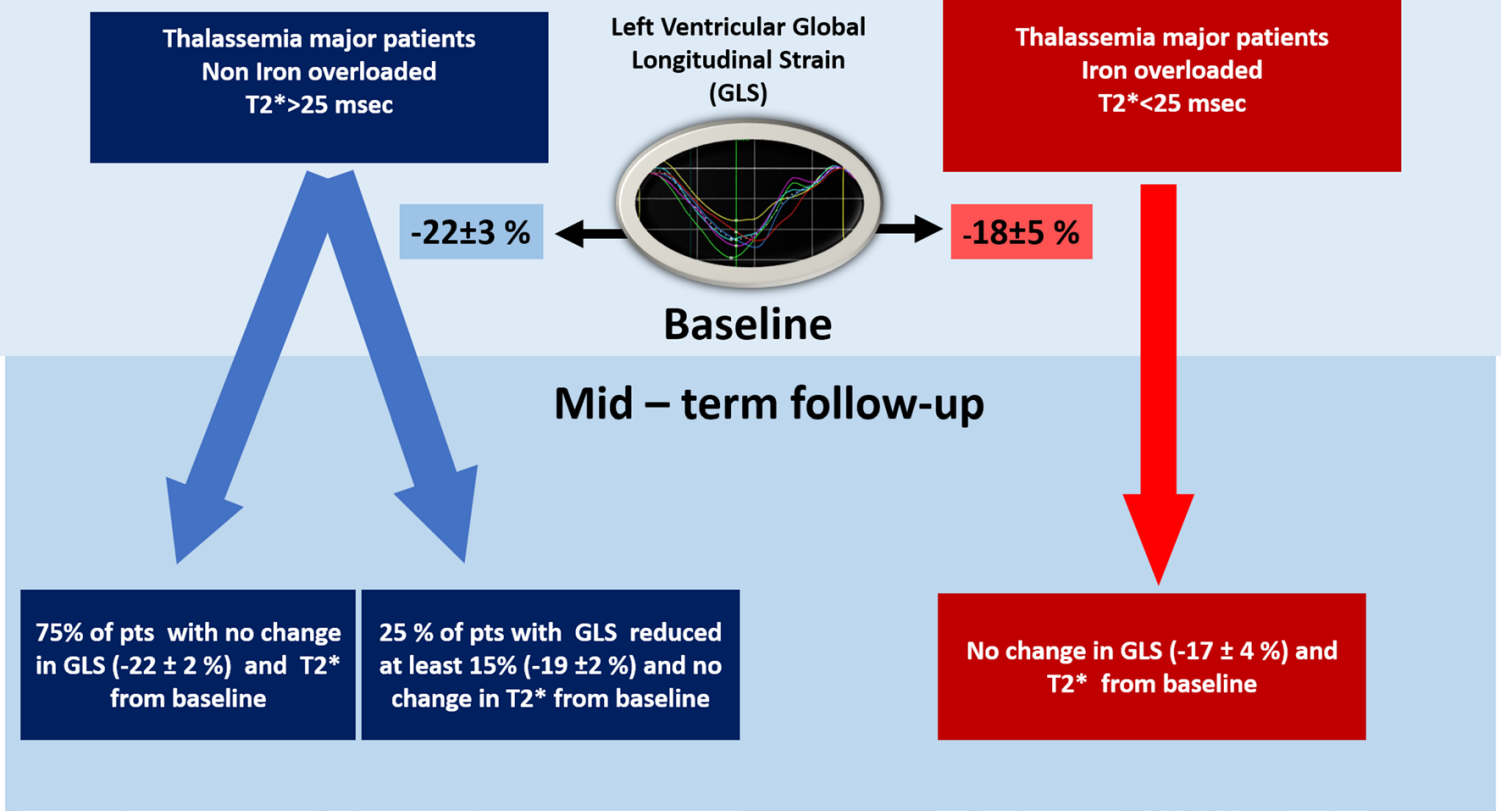
Left ventricular deformational mechanics in patients with and without myocardial iron overload at baseline and at midterm follow-up

We identified eight patients with GLS worsening. These non-iron–overloaded TM patients who exhibited GLS worsening during follow-up also had a marginally statistically significant deterioration in LVEF. There was no significant change in other echocardiographic parameters (Table 3) (Fig. 2).

**Table 3:** Characteristics of non cardiac iron overloaded patients, with or without a > 15% relative worsening in GLS during follow-up period

	< 15% relative worsening in GLS				> 15% relative worsening in GLS					
	Group 1a (24 pts)				Group 1b (8 pts)					
	Baseline	Follow-up	*p*	Δ (%)	Baseline	Follow-up	*p*	Δ (%)	Group 1a versus 1b Follow-up	Group 1a versus 1b Δ%
									*P*	*p*
T2* (msec)	34 ± 4	36 ± 3	0.12	6	34 ± 4	31 ± 5	0.40	− 8	0.06	0.12
Ferritin	1190 ± 1573	1295 ± 1998	0.64	43	674 ± 642	263 ± 103	0.15	− 39	0.25	0.18
*Echocardiographic data*										
Conventional										
LVEF (%)	62 ± 5	63 ± 3	0.47	2	65 ± 3	63 ± 3	0.046	− 3	0.75	0.09
LVEDD (mm)	50 ± 4	49 ± 4	0.06	− 2	47 ± 5	47 ± 6	1.00	0	0.54	0.37
LVESD (mm)	32 ± 4	31 ± 3	0.35	− 1	29 ± 4	30 ± 5	0.74	2	0.44	0.51
IVS (mm)	8.5 ± 0.9	8.6 ± 0.9	0.58	2	8.5 ± 1.2	8.9 ± 1.0	0.44	6	0.51	0.44
E/A	1.4 ± 0.3	1.4 ± 0.2	0.66	5	1.7 ± 1.1	1.5 ± 0.9	0.28	− 9	0.66	0.20
RVSm (cm/sec)	15 ± 1	15 ± 2	0.56	− 2	15 ± 3	16 ± 1	0.41	14	0.19	0.09
Deformational										
Circumferential strain (%)	− 21.0 ± 3.9	− 22.8 ± 5.4	0.15	9	− 21.9 ± 4.3	− 20.7 ± 6.1	0.63	− 5	0.66	0.21
Circumferential strain rate	− 1.2 ± 0.2	− 1.4 ± 0.4	0.10	18	− 1.3 ± 0.2	− 1.2 ± 0.1	0.10	− 14	0.86	0.06
Global Longitudinal strain (%)	− 21.3 ± 2.1	− 22.4 ± 2.2	0.05	6	− 24.5 ± 3.0*	− 19.2 ± 2.4	0.01	− 22	0.004	0.001
Mean Longitudinal strain rate	− 1.2 ± 0.2	− 1.3 ± 0.2	0.14	7	− 1.4 ± 0.3	− 1.0 ± 0.1	0.01	− 23	0.05	0.43

*LVEF* left ventricular ejection fraction, *GLS* left ventricular global longitudinal strain, *LVEDD* left ventricular end-diastolic diameter, *LVESD* left ventricular end-systolic diameter, *IVS* intraventricular septum thickness, *E/A* early to atrial trans-mitral flow ratio, *RVSm* right ventricular basal myocardial systolic velocity

^*^p = 0.02 compared to the corresponding value of Group1a at baseline

#### Correlation between various parameters at baseline and follow-up

There was a good correlation between LVEF and T2* and between LS and T2* values (Table 4). There was a significant correlation between liver and myocardium T2* at baseline but not in the follow-up measurements. There was a strong correlation between ferritin and liver T2* and a good correlation between ferritin and cardiac T2* at baseline but not at follow-up.

**Table 4:** Correlations between Cardiac T2*, mean serum Ferritin, Liver T2* and echocardiographic parameters, at baseline and follow-up of the study

	Baseline			Follow-up		
	Cardiac T2*	Ferritin	Liver T2*	Cardiac T2*	Ferritin	Liver T2*
LVEF						
Correlation	0.37	− 0.27	− 0.47	0.49	− 0.11	− 0.16
*P*	0.018	0.132	0.023	0.019	0.520	0.487
GLS						
Correlation	− 0.41	0.15	0.20	− 0.67	0.15	0.01
P	0.013	0.445	0.383	0.001	0.417	0.915
CS						
Correlation	− 0.14	0.27	0.50	− 0.1	0.15	0.13
*P*	0.411	0.195	0.036	0.716	0.341	0.593

CS: Mean Circumferential Strain (%), GLS: Global Longitudinal Strain (%), LVEF: Left Ventricular Ejection Fraction (%)

## Discussion

The main findings of the present study suggest that in patients with TM, left ventricular longitudinal strain (GLS) can detect LV subclinical dysfunction due to high cardiac iron load better than LVEF. There was a good correlation of cardiac T2* with both LVEF and GLS, both at baseline and follow-up. At the mid-period of follow-up, we detected no changes in conventional or deformational echocardiographic parameters in either iron– or non-iron–overloaded TM patients. Interestingly, during the follow-up period, we detected a relative worsening in the left ventricular longitudinal strain by more than 15%, with a concomitant marginal reduction in their corresponding LVEF, in eight patients in the non-iron—overloaded patient group.

Two-dimensional and three-dimensional LVEF measurement using echocardiography is the standard method for evaluating systolic function in TM patients. Serial studies have identified that a reduction in LVEF ≥ 7% is a strong predictive factor for cardiac death in TM populations [14, 15]. Once heart failure symptoms occur, survival decreases substantially [16]. The central role of iron-mediated cardiac toxicity in TM patients established the role of CMR for cardiac iron load quantification, guiding the usage of iron chelator therapy and thus improving patient outcome [17, 18].

Early impairment of other indices of left ventricular systolic function using tissue Doppler imaging, before any remarkable reduction in left ventricular ejection fraction, has already been reported [16, 19, 20]. In this direction, echocardiographic deformational analysis [21] in TM patients aims to detect early changes in cardiac mechanics before any observed reduction in LVEF. This strategy could potentially prompt a change in the patient’s therapeutic strategy. Previous echocardiographic studies have reported mixed results in correlating T2* values with cardiac deformational parameters. Monte et al. [22] and Li et al. [23] found no correlation between left ventricular deformational parameters and T2* signals. Our study is in agreement with others [24, 25], where they found a correlation of longitudinal strain with the T2* signal. It is noteworthy that in our study, iron–overloaded patients had similar LVEFs compared to non-iron–overloaded patients. Furthermore, a previous study revealed that cardiac and liver iron–overloaded patients have impaired left ventricular global longitudinal strain compared to non-iron–overloaded patients [25]. Future studies could evaluate whether T2* signal measurements combined with deformational parameters obtained by cardiac MRI can potentially provide better risk stratification for TM patients. Particularly in young patients, the identification of myocardial fibrosis by MRI could be evidence of previous iron overload episodes.

During the follow-up period, iron load status according to cardiac T2* did not change in either iron– or non-iron–overloaded patients; accordingly, we did not find significant changes in conventional or deformational echocardiographic parameters, while a statistically significant correlation between T2* signal and GLS remained. This suggests that left ventricular deformational analysis can detect early myocardial systolic dysfunction and could be a sensitive tool for serial follow-up measurements of cardiac function. The absence of improvement in deformational parameters and T2* values during follow-up in iron–overloaded patients could be attributed to the patient’s compliance issues with chelation therapies, and irreversible myocardial damage secondary to repeated exposure of the myocardium to toxic factors related to thalassemia major may also play a role.

In our study, circumferential strain (CS) was also measured as an additional index of myocardial deformation, but we identified no correlation between CS and CMR T2* signals. Previous studies have shown that GLS is a more sensitive parameter than CS in identifying early impairment of LV function [26, 27].

Impairment of left ventricular longitudinal strain has been identified as an early marker of left ventricular dysfunction in patients undergoing chemotherapy [11]. In our study, in the group of non-iron–overloaded patients, we identified 8 of 32 patients who revealed a more than 15% worsening in their left ventricular LS deformational parameter with a marginal reduction in the EF. Consistent with our findings, the results from Marsella et al. [28] reported that up to 10% of TM patients without cardiac iron overload could still develop heart failure.

The pathogenesis of systolic dysfunction in TM is complex. Apart from the toxic effect of iron load, there is a significant contribution of the immunoinflammatory and inherited components [29–31]. Chronic tissue hypoxia in addition to chronic anemia, nutritional deficiencies and viral infections may contribute to the different susceptibilities to iron overload and cardiac damage. Moreover, myocardial fibrosis has been revealed in CMR studies in both iron– and non-iron–overload patients [32]: long-lasting consequences of previous damage could explain the contradictory results on the correlation between the actual CMR T2* signal and left ventricular deformational parameters [22–25, 32]. Our results demonstrated a significant correlation between liver and heart iron load assessed by MRI-T2*, while this correlation was lost at follow-up. These contradictory findings are consistent with previous reports [33, 34]. These results could be attributed to differences in iron transport and deposition in these organs [33]. Furthermore, we identified good correlations between ferritin and myocardial and liver T2* measurements. However, during follow-up, the ferritin-liver T2* correlation was persistent, but the ferritin-heart T2* correlation was not. Other studies have also reported contradictory findings in the correlation between ferritin and iron deposition on the heart and liver measured by MRI [35–37], indicating that serum ferritin cannot reliably predict the liver and heart iron content (Table 4).

The small number of patients in our study indicates that our results need validation with a larger group. Additionally, longer and less variable follow-up periods could potentially detect subtle differences in the deformational mechanics in patients with thalassemia major myocardial iron overload. The cutoff point value of a 15% reduction in GLS (validated in other patient groups) is arbitrary in patients with TM, and more studies are needed to define its clinical significance. Finally, late enhancement studies with gadolinium are not performed routinely during CMR to search for myocardial fibrosis. These studies could further characterize the myocardium in TM patients.

## Conclusions

The present study suggests an important role of echocardiography in TM patients, identifying early left ventricular dysfunction with the use of longitudinal strain parameters. TM patients with high iron cardiac load had low left ventricular longitudinal deformation, although LVEF values were normal. For a midterm follow-up period, there was no significant change in myocardial deformational parameters parallel to the absence of T2* value changes in patients with TM. Additionally, in a small subgroup of patients without myocardial iron overload, a deterioration in left ventricular deformational parameters was observed before a striking decrease in LVEF. These findings emphasize the significant contribution of serial GLS measurements on top of MRI follow-up, aiming to identify those patients with myocardial dysfunction at early stages, prompting potential therapies.

## Data Availability

The datasets used and/or analyzed during the current study are available from the corresponding author on reasonable request.

## Abbreviations

CS: Circumferential strain
GLS: Global longitudinal strain
LV: Left ventricle
LVEF: Left ventricular ejection fraction
LS: Longitudinal strain
MRI: Cardiac magnetic resonance Imaging
RVSm: Right ventricular systolic myocardial velocity by tissue Doppler
TM: Thalassemia major
